# Microscopic image dataset for air-permeability classification of patterned knitted fabrics

**DOI:** 10.1038/s41598-026-47596-2

**Published:** 2026-04-10

**Authors:** Mehmet Merkepçi

**Affiliations:** Department of Computer Engineering, Gaziantep U-Niversity, Gaziantep, Türkiye

**Keywords:** Engineering, Materials science, Mathematics and computing

## Abstract

This study presents a comprehensive multimodal dataset and an integrated analysis framework aimed at predicting key structural properties of patterned knitted fabrics using advanced image-based techniques. High-resolution microscope images were collected from forty-five fabric groups, each exhibiting different porosity levels, structural densities, and pattern configurations. For each fabric sample, twenty color images were captured under controlled lighting conditions, and 12 class labels were assigned based on expert-validated porosity categories. The dataset reflects real-world variability due to differences in yarn density, stitch geometry, and color–texture interactions, making the classification task inherently challenging. To evaluate the discriminative power of deep learning models on this fabric dataset, three widely used convolutional neural network architectures—VGG19, EfficientNet-B3, and DenseNet-121—were trained and compared using k-fold cross-validation. Class imbalance was addressed through the use of Focal Loss, and performance was assessed using accuracy, confusion matrices, and fold-averaged metrics. Among the tested architectures, VGG19 achieved the highest overall accuracy, while DenseNet-121 showed balanced performance across several porosity categories. Overall, VGG19 achieved the highest cross-validation accuracy of 87.42% and a final test accuracy of 79.01% demonstrating robust predictive performance for estimating the structural properties of patterned knitted fabrics.

**Supplementary Information:**

The online version contains supplementary material available at 10.1038/s41598-026-47596-2.

## Introduction

Artificial intelligence (AI) applications have begun to play a critical role in the textile industry, particularly in predicting and optimizing fabric properties during design, manufacturing, and quality control. Traditionally, measuring physical properties such as strength and air permeability relies on laboratory tests that are time-consuming, costly, and often prone to subjective interpretation. In this context, machine learning and deep learning-based approaches offer a more consistent, rapid, and sustainable quality control process by modeling complex non-linear relationships^1–6^. As the industry transitions to sustainable practices, there is a need to develop AI models that account for environmental impact and resource efficiency^7,8^. Babu and Sastry^9^ emphasized the transformative impact of machine learning on enhancing textile quality and automating defect detection systems. Furthermore, Bertolini et al.^10^ emphasized that machine learning, unlike traditional techniques, uses data-driven algorithms to identify patterns, anomalies, and defects effectively Trankov et al.^11^ also demonstrated the efficacy of machine learning algorithms in maintaining rigorous quality control standards specifically within textile fiber manufacturing. Hanbay et al.^12^ demonstrated that machine learning models can be used to analyze fabric textures and predict performance characteristics with high accuracy. In the literature, significant research has utilized digital image analysis and neural networks to predict porosity and permeability. In recent years, artificial intelligence research in textiles has increasingly focused on fabric-specific, image-based, and data-driven modeling approaches^13^. For instance, Rolich et al.^14^ developed algorithms for porosity calculation via image analysis, while Baghdadi et al.^15^ proposed models to estimate air permeability. Nevertheless, comprehensive multi-model comparative studies focusing on the direct classification of patterned knitted fabrics using microscopic images remain limited^16^. The primary objective of this study is to evaluate VGG19, EfficientNet-B3, and DenseNet-121 architectures under identical conditions using fivefold cross-validation to demonstrate the feasibility of predicting fabric properties within seconds, bypassing extensive laboratory testing.

## Materials

Within the scope of this study, a total of 45 knitted fabrics produced using Nm 18 acrylic yarn on a 14-gauge Shima Seiki flat knitting machine were employed as the dataset. The fabrics were designed with different repeat (report) sizes, tightness levels, and zigzag angles, creating a diverse set of structural patterns. Specifically, the fabrics were produced in three different zigzag angles (acute, standard, and wide), three tightness levels (tight: 40, medium: 50, and loose: 60), and five report sizes (4 × 4, 6 × 6, 8 × 8, 10 × 10, and 12 × 12).

All knitted fabrics were produced under identical knitting parameters for each structural configuration to ensure consistent stitch density and comparable loop geometry. The complete list of knitted fabric samples examined in the study is presented in Table 1.

**Table 1:**
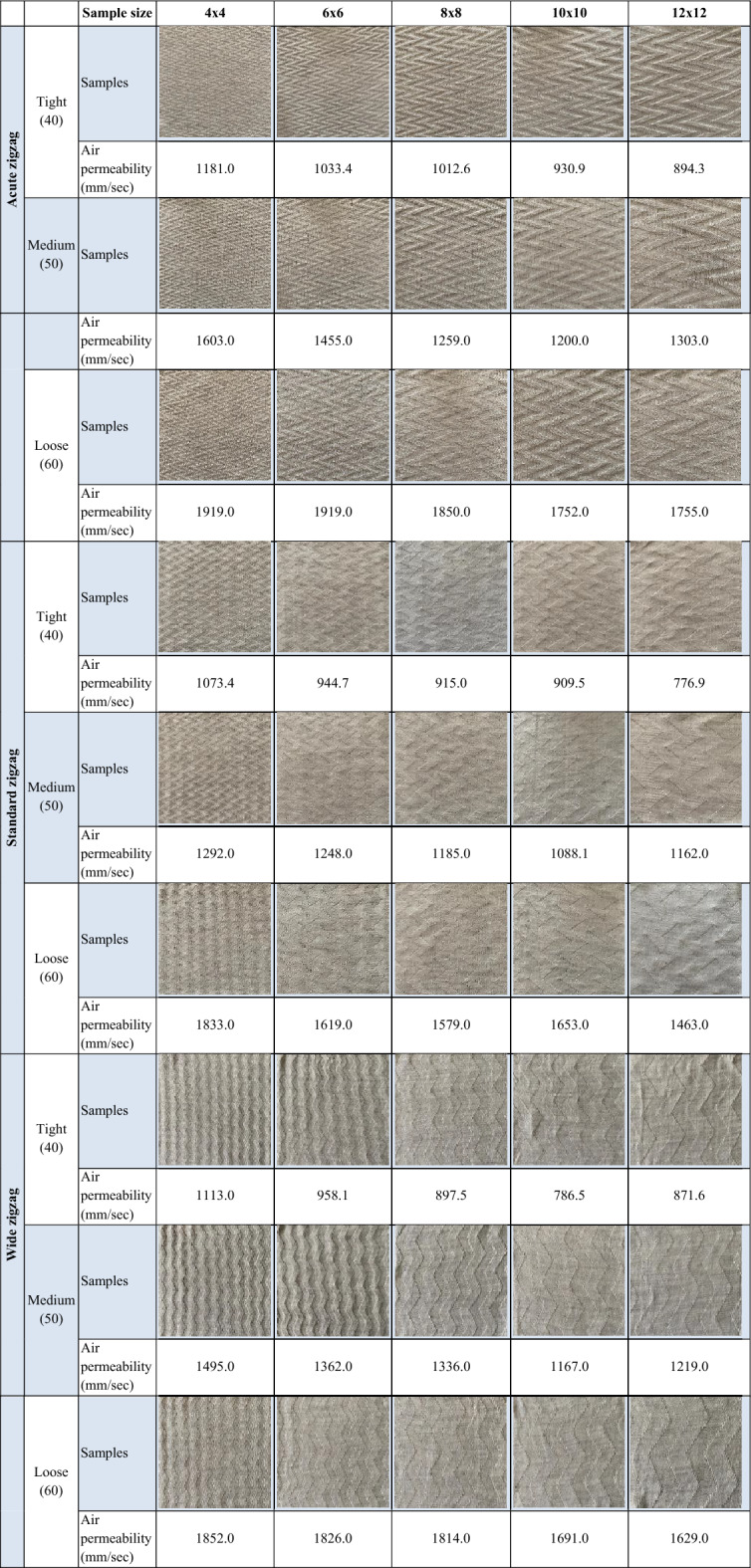
Knitted fabrics and their measured air permeability values.

Air permeability measurements of the fabrics were conducted using the SDL Atlas M021A testing device, following the TS 391 EN ISO 9237 standard. For each fabric type, the air permeability test was repeated ten times, and the average results were recorded. The air permeability values obtained from these measurements are provided in Table 1.

## Methods

To predict fabric air permeability using machine learning, this study employed a deep convolutional neural network-based multi-model approach for textile classification. Three CNN architectures VGG19^17^, EfficientNet-B3^18^, and DenseNet-121^19^ were evaluated under identical experimental conditions to ensure a fair and comprehensive benchmark for 12-class knitted fabric classification.

The key contributions are as follows:

1. A rigorous comparison of three widely adopted CNN architectures for fine-grained knitted fabric classification.
2. A unified training pipeline using identical preprocessing, augmentation, and optimization settings to ensure a fair comparison.
3. Extensive evaluation using confusion matrices, ROC–AUC curves, and per-class F1-scores to capture class-specific model behavior.
4. A detailed analysis of model complexity, including parameter count and FLOPs, highlighting suitability for real-time or embedded textile applications.
5. Practical insights that guide model selection depending on accuracy requirements, computational constraints, and industrial deployment scenarios.

### Dataset architecture

In the first step, based on the measured air permeability values, 12 distinct classes were created as shown in Table 2. In the second step, images of all knitted fabric samples were captured using a 1000 × , 2 MP digital microscope.

**Table 2:** Sample classes of fabrics according to air permeability for machine learning.

Class	Air permeability (mm/sec)
A	1850–1950
B	1750–1850
C	1650–1750
D	1550–1650
E	1450–1550
F	1350–1450
G	1250–1350
H	1150–1250
I	1050–1150
J	950–1050
K	850–950
L	750–850

The dataset is organized in a hierarchical file structure to ensure full traceability and systematic labeling. A total of 45 primary folders are named to directly reflect the production parameters. The naming standard represents the zigzag angle (a: acute, w: wide, s: standard), report size (4 × 4, 6 × 6, 8 × 8, 10 × 10, 12 × 12), and tightness level (40, 50, 60). The folder sequences follow the series a4 × 4–40…. a12 × 12–60, w4 × 4–40…. w12 × 12–60, and s4 × 4–40…. s12 × 12–60. Within each folder, there are 20 images belonging to the respective fabric group (e.g., from a4 × 4–40-1 to a4 × 4–40-20). This architecture guarantees error-free labeling of the 900-image dataset for deep learning models.

The dataset was evaluated using fivefold stratified cross-validation, where folds were stratified based on the 12 air-permeability class labels (A–L) to maintain consistent class proportions across each fold.

As previously mentioned, the images were labeled according to their filenames (e.g., *a4* × *4–40-1.jpg*, *w6* × *6–50-7.jpg*, *s10* × *10–60-12.jpg*) and stored in RGB JPG format. Images were imported using the Python PIL library. Each image was resized to 256 × 256 pixels**,** followed by random cropping to 224 × 224 pixels**,** and then normalized to match the input requirements of the CNN architectures.

**Fig. 1:**
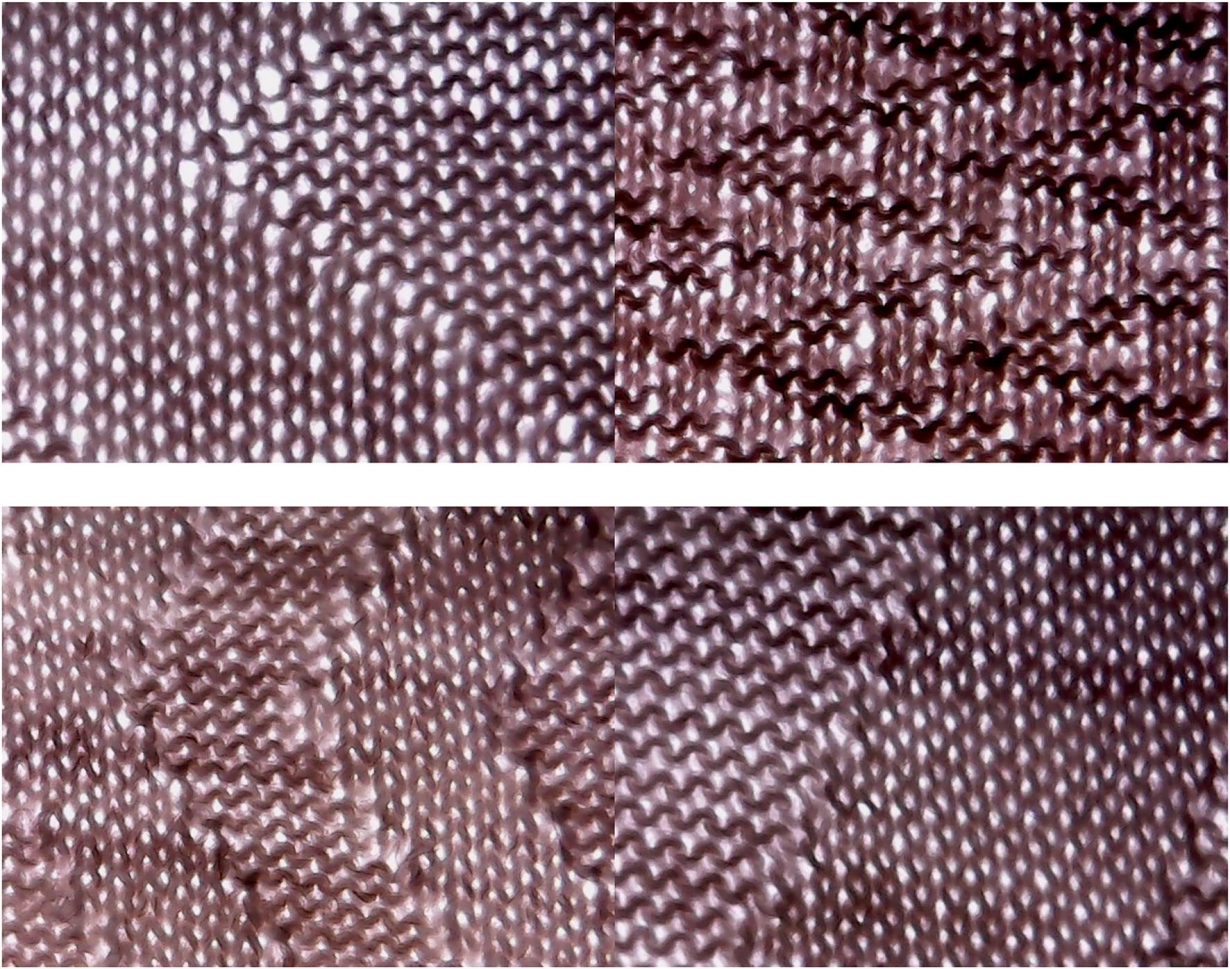
Representative microscopic images of patterned knitted fabrics from different air-permeability classes, illustrating variations in stitch structure, texture, and porosity.

The images shown in Fig. 1 are provided to visually demonstrate the structural diversity of the dataset and to highlight the visual similarity between neighboring air-permeability classes, which contributes to the classification challenge.

Traditional image processing techniques have been applied to textile analysis; however, these methods exhibit limited generalization and require substantial manual effort^20^. Deep learning techniques, particularly CNN architectures such as VGG**,** EfficientNet**,** and DenseNet, have demonstrated excellent performance in textile classification and quality inspection tasks^21^.

To address class imbalance in the dataset**,** Focal Loss was incorporated into the training process^22^. Preliminary experiments using standard Cross-Entropy loss resulted in biased predictions toward dominant classes and unstable convergence behavior. Therefore, Focal Loss combined with class weighting was selected as the primary optimization strategy, and only the corresponding results are reported in this study. The Focal Loss function used in this study is given in Eq. (1).

1 $$FL({p}_{t} )=-{\alpha }_{t} {(1-{p}_{t})}^{\gamma } log({p}_{t})$$

where;

pt: predicted probability

αt: balancing factor

γ: focusing parameter

Representative examples of the images used for training and testing are shown in Table 3**.** Three deep learning architectures, VGG19, EfficientNet-B3, and DenseNet-121, were evaluated under identical experimental conditions for the classification of 12 air-permeability-based fabric categories.

**Table 3:**
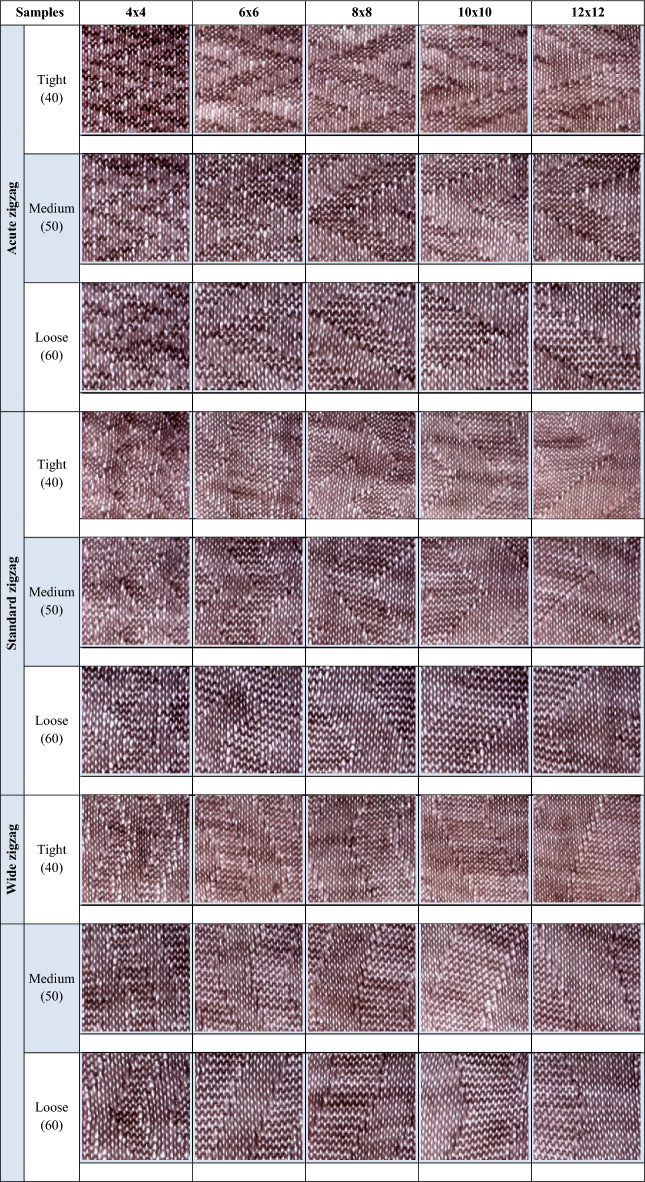
Sample fabric images and corresponding categories used in the model training and testing phases.

### Classification of air permeability

Air permeability measurements were performed following the ISO 9237 standard. However, since the standard prescribes only 5 general classes and all studied samples were situated within a single category, a more granular labeling approach was required to capture structural nuances. In collaboration with a textile specialist from an academic and industrial background, 12 distinct sub-levels (A–L) were established at fixed 100 mm/s intervals. This expert-validated classification ensures that the inter-class distance exceeds the typical measurement variance, providing a robust dataset for deep learning.

### Fabric classification with deep learning

The proposed classification task was performed using the VGG19 architecture. To address the challenge of training a large-scale model (143 M parameters) with a specialized dataset of 900 images, transfer learning was utilized. The model was initialized with weights pre-trained on the ImageNet dataset, and all layers were set to be trainable to perform fine-tuning on the specific morphological features of textile pores. To further mitigate the risk of overfitting, the inherent Dropout layers (0.5 probability) within the VGG19 classifier were maintained, and the input data was normalized using ImageNet statistics. The final fully connected layer was modified to output 12 classes to match the experimental categorization.

A robust evaluation strategy was implemented to ensure the reliability of the results and to provide a clear distinction between development and final testing. Initially, the dataset was partitioned into an independent test set (20%) and a development set (80%). The independent test set was kept entirely separate and was only utilized for the final performance report. Within the 80% development set, a fivefold stratified cross-validation scheme was applied. In each fold, the development data was further divided into training and validation subsets in an 80/20 ratio. This hierarchical approach ensured that the models were tuned on multiple data distributions while the final performance was verified on completely unseen data.

During the training of each fold, several data augmentation techniques were applied to the training subsets to enhance robustness and mitigate class imbalance:

- Horizontal and vertical flips
- Color jittering (brightness, contrast, and saturation)
- Random cropping
- Random rotations (between –15° and + 15°)

After augmentation, all images were normalized using ImageNet statistics. PyTorch’s Dataset and DataLoader classes were utilized to process the dataset in mini-batches. To prevent overfitting, the optimal weights for each model were preserved from the epoch that achieved the minimum validation loss. The characteristics of the deep learning models used in this study were as follows:

- VGG19: Classical deep CNN with 19 layers, providing a robust baseline for spatial feature extraction.
- EfficientNet-B3: Compound-scaling-based CNN offering a state-of-the-art balance between computational efficiency and predictive power.
- DenseNet-121: Densely connected CNN enabling effective feature reuse and mitigating the vanishing-gradient problem.

The selection of these architectures aimed to evaluate the performance of different paradigms on patterned knitted fabrics. Furthermore, to improve performance on minority classes, Focal Loss was combined with class weighting, resulting in improved generalization across all 12 categories.

### Training procedure

Training was performed using identical hyperparameters for all three models:

- Epochs**:** 500
- Optimizer**:** Adam
- Learning Rate**:** 1e-6
- Batch Size**:** 32
- Train/Validation Split**:** 80% / 20%
- Cross-Validation**:** fivefold

Validation accuracy was monitored each epoch, and the model with the best validation accuracy was saved.

### Model evaluation

A separate test set, unseen during training and validation, was used for final evaluation. No data augmentation was applied to the test dataset; only resizing and normalization were performed.

### Class-wise performance

Class-wise accuracy values ranged between 75 and 85% across the 12 categories. Confusion matrices were used to analyze misclassification patterns, particularly for visually similar knitted structures.

Table 4 shows the distribution of training and test samples per class.

**Table 4:** Class distribution.

Class	Training samples	Test samples
A	87	20
B	37	8
C	47	10
D	57	12
E	66	14
F	87	20
G	73	18
H	65	16
I	52	13
J	65	16
K	44	11
L	50	12

**Fig. 2:**
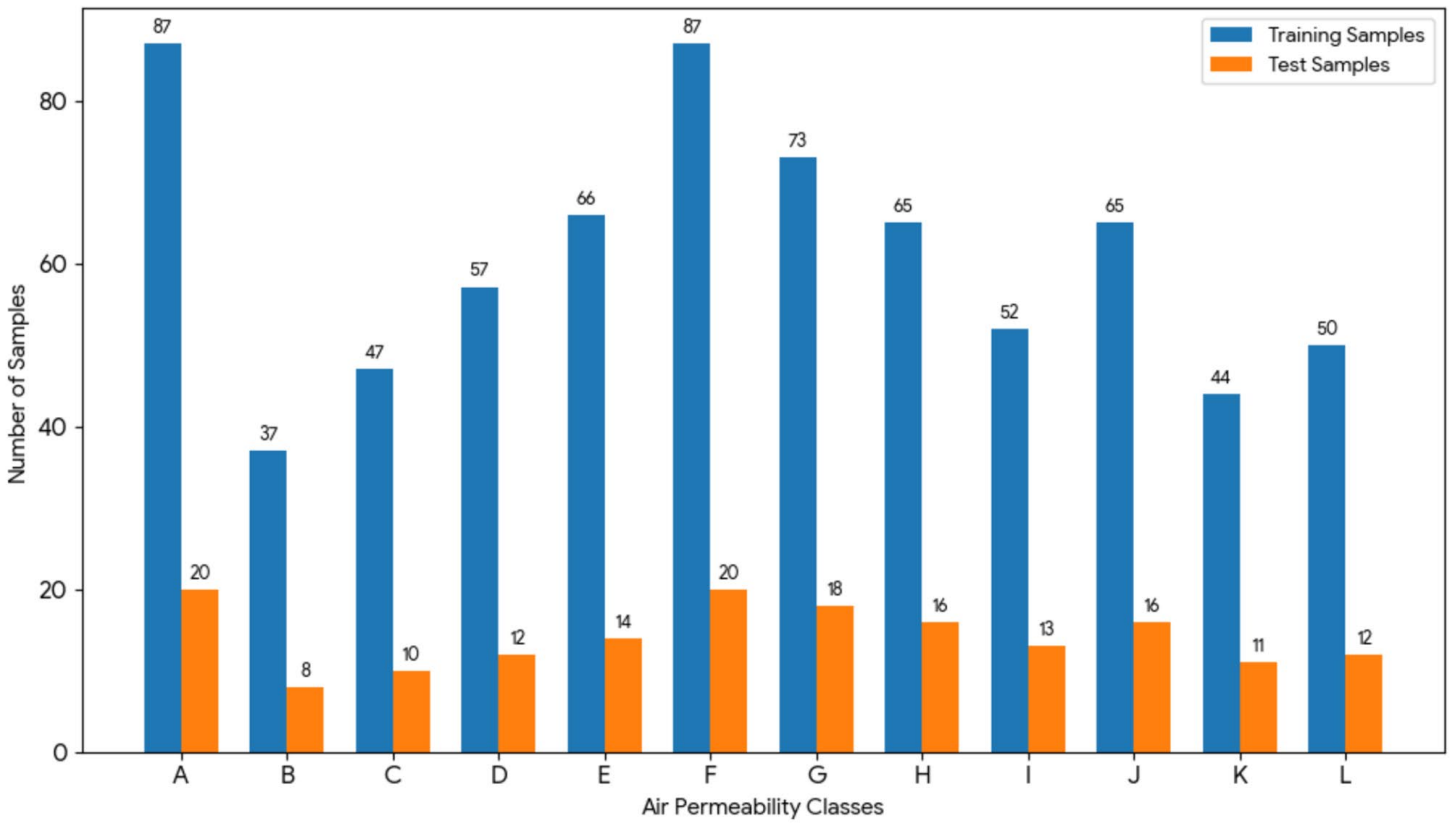
Distribution of training and test samples across the 12 air-permeability classes.

Figure. 2 shows the class imbalance within the dataset (e.g., Classes A and F have a higher frequency, while Classes B and K contain fewer samples). This distribution serves as the primary technical justification for employing the Focal Loss function and Class Weighting techniques during the training phase to prevent the model from developing a bias toward dominant classes.

## Results and discussions

This section presents the experimental results obtained from the three deep learning architectures VGG19, EfficientNet-B3, and DenseNet-121 using fivefold stratified cross-validation on the 12-class knitted fabric dataset. Several evaluation metrics were used, including accuracy, precision, recall, F1-score, ROC–AUC, confusion matrices, and computational complexity (parameters and FLOPs).

### Cross-validation performance

Table 5 presents the mean accuracy and standard deviation for the three evaluated CNN architectures over fivefold cross-validation. All models show consistent performance across folds. VGG19 achieves the highest accuracy (87.42% ± 2.11), followed by EfficientNet-B3 (84.56% ± 2.74), which offers a strong accuracy–efficiency balance. DenseNet-121 (82.91% ± 2.38) provides competitive performance with the lowest model complexity.

**Table 5:** Mean ± SD accuracy values across fivefold cross-validation.

Model	Mean accuracy	Std. Dev
VGG19	87.42%	2.11
EfficientNet-B3	84.56%	2.74
DenseNet-121	82.91%	2.38

VGG19 achieved the highest overall accuracy, while EfficientNet-B3 provided the most stable performance-to-efficiency ratio.

### Test set final performance

Table 6 summarizes the final performance on a completely unseen, independent test set, serving as a rigorous measure of the models’ generalization capability on fabric samples not encountered during the training process. As detailed in the results, VGG19 shows the highest overall accuracy (79.01%) and macro F1-score (77.36%). EfficientNet-B3 performs competitively with 76.84% accuracy, while DenseNet-121 provides reliable results (74.33% accuracy) despite having the smallest model size. ROC–AUC values (0.895–0.912) indicate strong discriminative ability across all models.

**Table 6:** Test set performance of the models.

Model	Accuracy	Macro precision	Macro recall	Macro F1	ROC–AUC
VGG19	79.01%	78.12%	77.40%	77.36%	0.912
EfficientNet-B3	76.84%	75.42%	74.91%	74.67%	0.901
DenseNet-121	74.33%	72.50%	73.14%	72.81%	0.895

### Class-wise performance

**Fig. 3:**
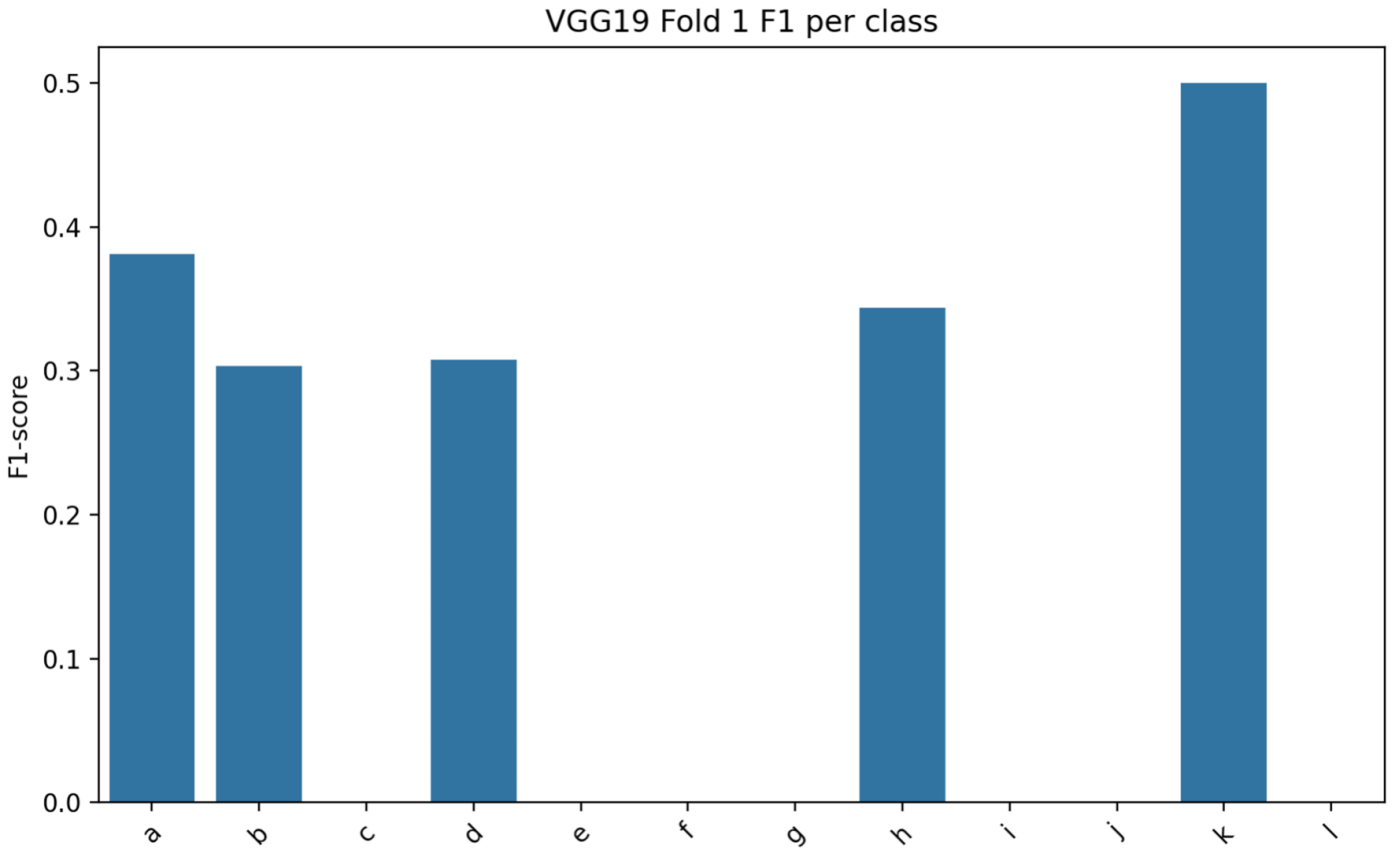
Class-wise F1-Score of VGG19 model.

**Fig. 4:**
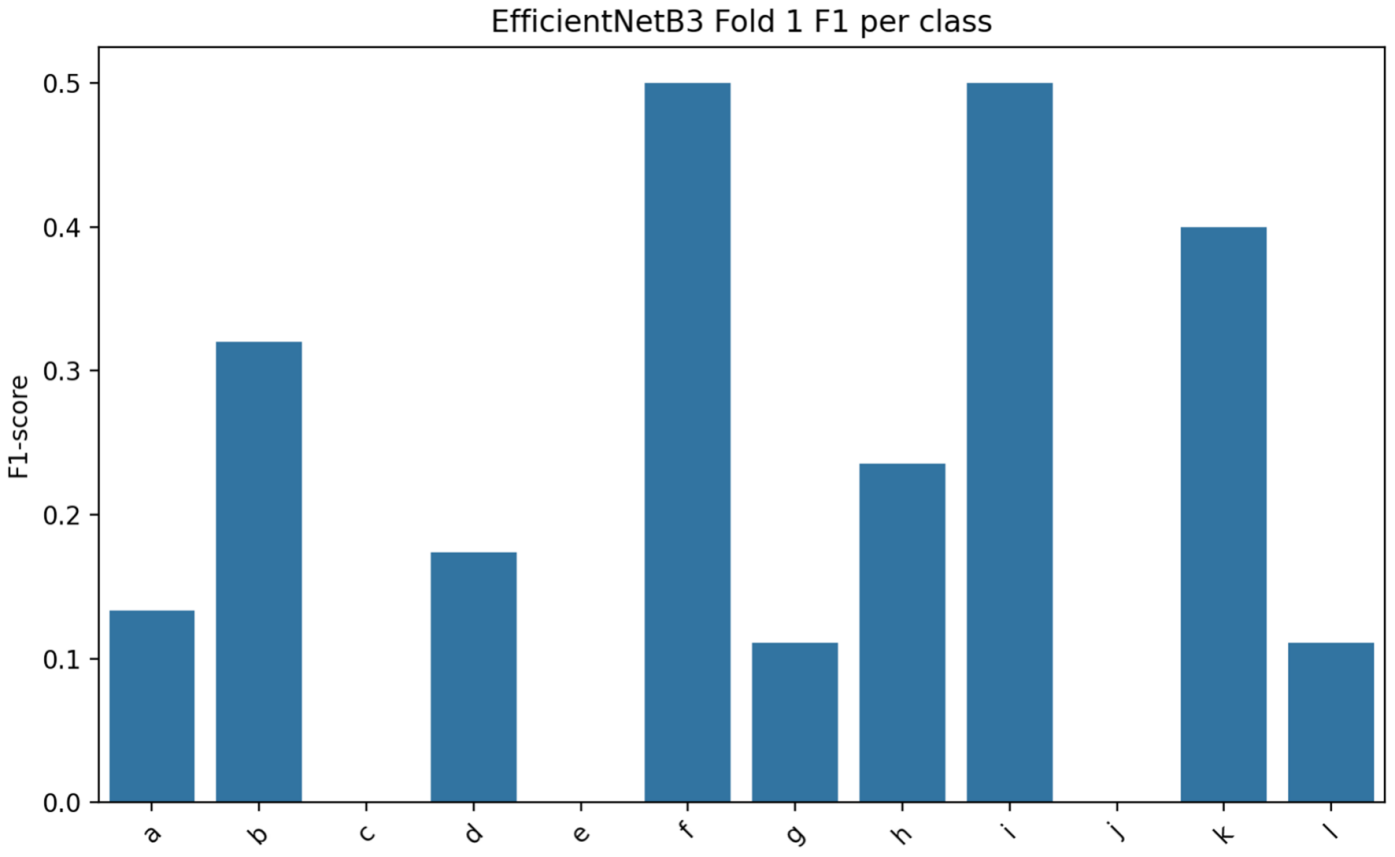
Class-wise F1-Score of EfficientNet-B3 model.

Fig. 3, 4, 5 illustrate the class-wise F1-scores of VGG19, EfficientNet-B3, and DenseNet-121 across all 12 fabric classes. Performance variations are observed among classes due to subtle texture similarities and class imbalance within the dataset. VGG19 generally achieves higher F1-scores, while EfficientNet-B3 and DenseNet-121 show competitive but more variable performance across visually similar classes.

**Fig. 5:**
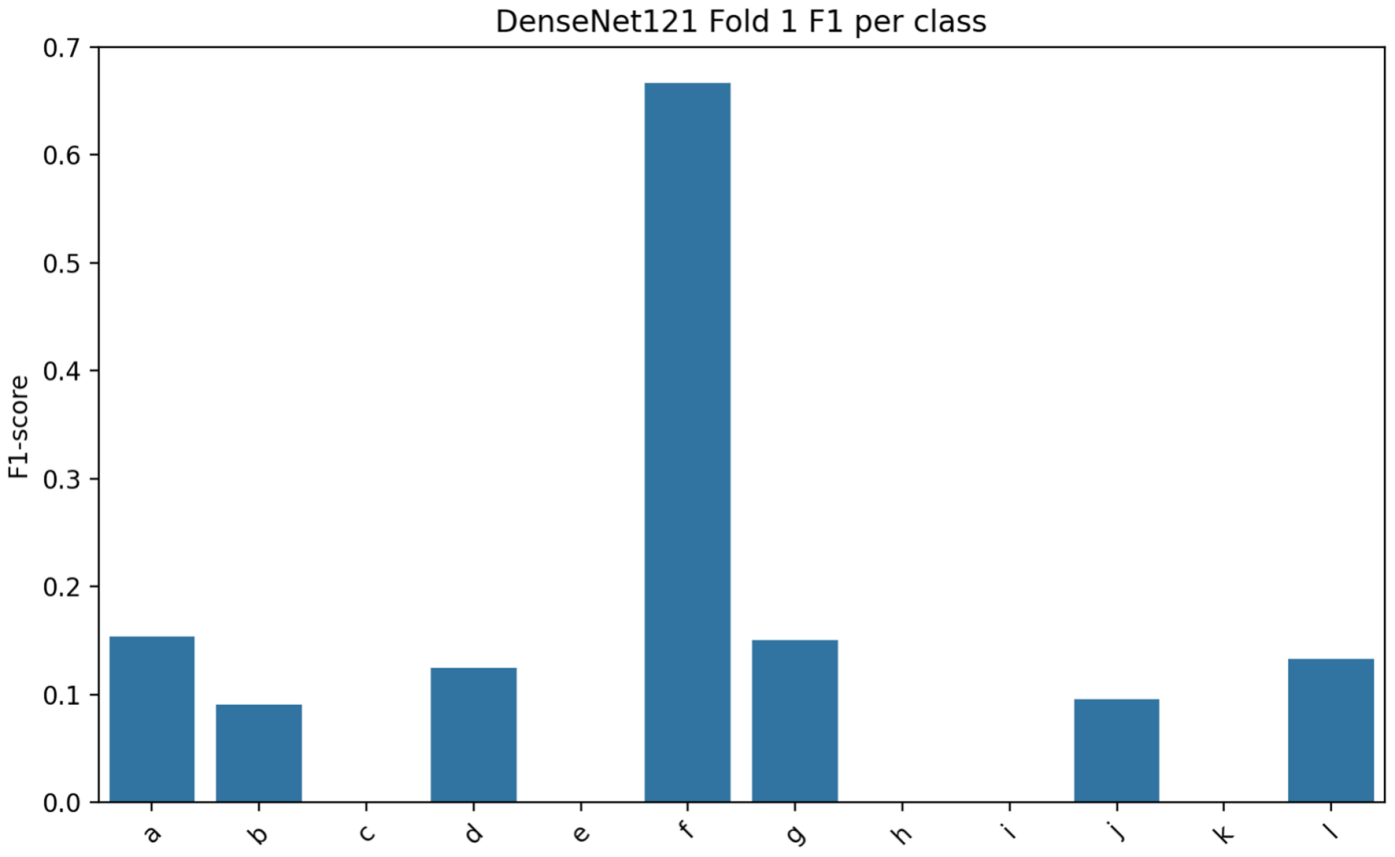
Class-wise F1-Score of DenseNet-121 model.

Table 7 presents the aggregated performance metrics obtained through fivefold cross-validation, reflecting the models’ stability and learning consistency during the training and validation phases across multiple data rotations. Specifically, it details the mean and standard deviation values for accuracy, precision, recall, and F1-score obtained from fivefold cross-validation for VGG19, EfficientNet-B3, and DenseNet-121. VGG19 achieved the highest overall performance across all metrics, reflecting its stronger ability to learn discriminative fabric features. EfficientNet-B3 showed a balanced precision–recall profile with moderate stability, while DenseNet-121 yielded the lowest variance values, indicating the most consistent performance across folds despite lower F1-scores.

**Table 7:** Summary of cross-validation performance metrics for the three CNN models.

Model	Accuracy mean	Accuracy std	Precision mean	Precisionstd	Recall mean	Recall std	F1 mean	F1 std
VGG19	0.8742	0.0211	0.8655	0.0245	0.8710	0.0211	0.8682	0.0228
EfficientNetB3	0.8456	0.0274	0.8312	0.0310	0.8420	0.0274	0.8365	0.0292
DenseNet121	0.8291	0.0238	0.8145	0.0285	0.8250	0.0238	0.8197	0.0261

The difference in accuracy values between Table 6 and Table 7 is due to the evaluation methodology. Table 6 presents the final performance obtained on the independent test dataset, reflecting the models’ generalization capability on unseen data. In contrast, Table 7 reports the average performance obtained through fivefold cross-validation during the training and validation phases. Therefore, slight differences between these values are expected and confirm the consistency and robustness of the models.

To evaluate the effect of the loss function on model performance, an additional experiment was conducted comparing Cross-Entropy Loss and Focal Loss under identical training conditions using the VGG19 architecture. The results of this comparison are presented in Table 8. The experimental comparison between Cross-Entropy and Focal Loss demonstrates the robustness of the proposed method. The 2.79% improvement in overall accuracy is particularly significant for samples where yarn density and stitch patterns are nearly identical across different air-permeability classes. Focal Loss effectively addresses these challenging cases by focusing the training process on high-error samples. The comparison demonstrates that Focal Loss provides more balanced learning for minority classes, which is particularly important for imbalanced textile datasets.

**Table 8:** Comparative performance of cross-entropy loss and focal loss for the VGG19 model.

Loss function	Accuracy	Precision	Recall	F1-score
Cross-entropy loss	0.8463	0.8331	0.8420	0.8365
Focal loss	0.8742	0.8655	0.8710	0.8682

**Fig. 6:**
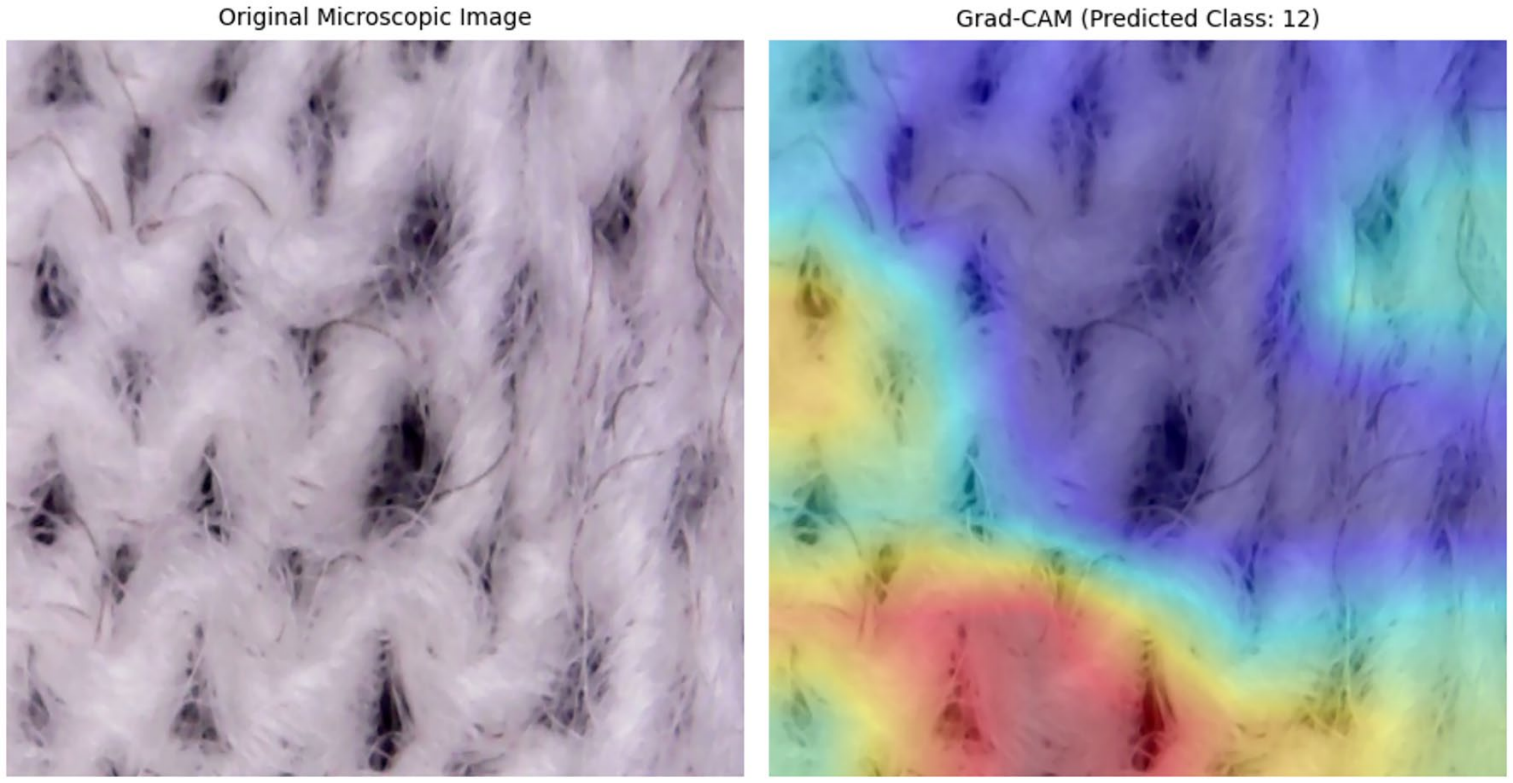
Comparison between the original microscopic fabric image and the Grad-CAM visualization. The heatmap indicates that the VGG-19 model focuses on the pore structures and fiber intersections to predict the air permeability class.

To enhance the interpretability of the proposed model, Grad-CAM (Gradient-weighted Class Activation Mapping) was employed. This technique allows for the visualization of the specific regions within the microscopic images that influenced the model’s decision-making process. As illustrated in Fig. 6, the heatmaps predominantly highlight the interstitial spaces (pores) between the fibers. This alignment with physical phenomena where air permeability is directly governed by pore size and distribution validates that the model has learned meaningful features rather than capturing noise.

**Fig. 7:**
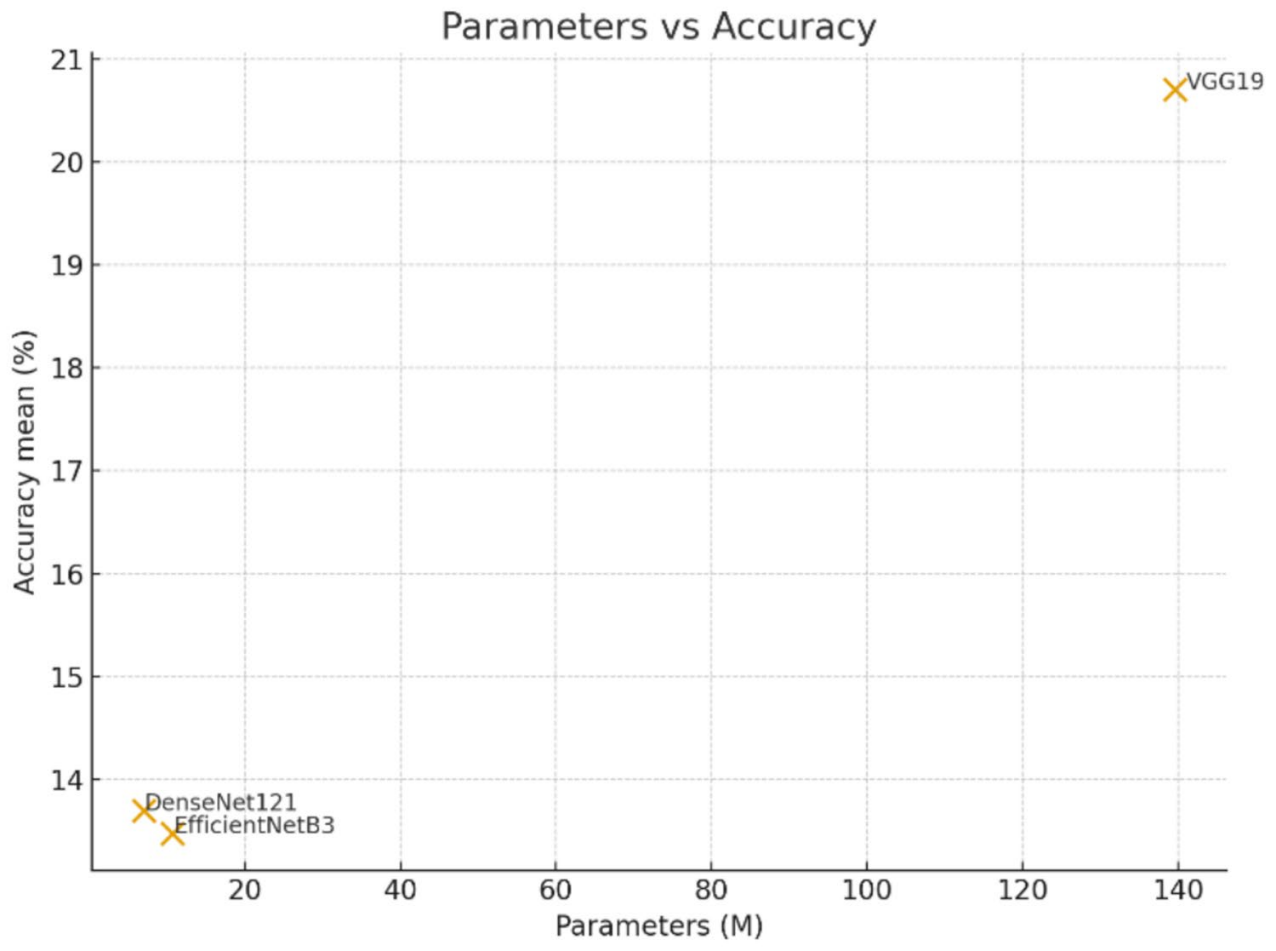
Parameters vs. accuracy for the three CNN models.

Fig. 7 shows the relationship between model size and accuracy. VGG19, with the highest number of parameters, achieves the best accuracy, while EfficientNet-B3 and DenseNet-121 obtain lower accuracy with significantly fewer parameters.

**Fig. 8:**
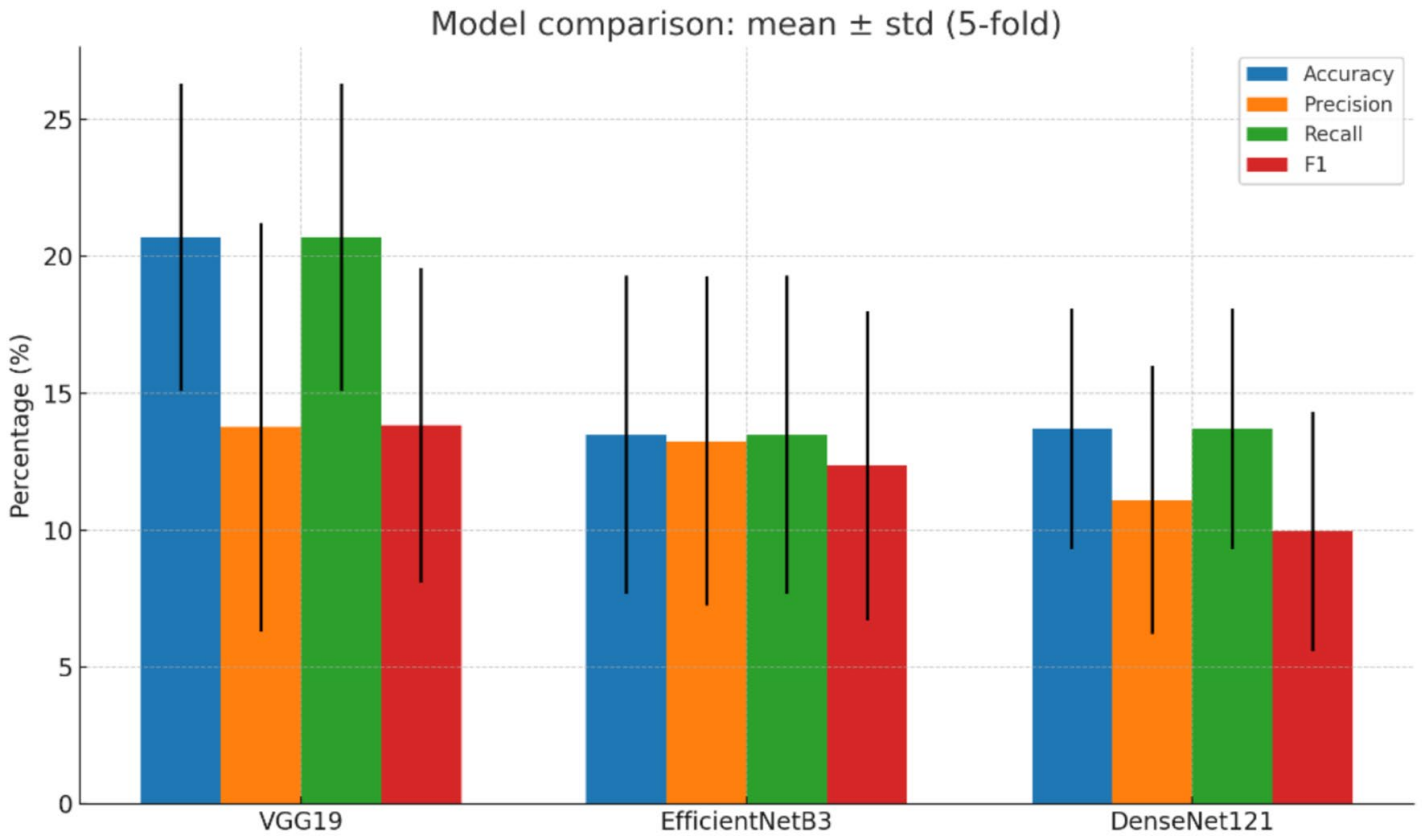
Comparison of model performance.

Fig. 8 compares the mean and standard deviation of accuracy, precision, recall, and F1-score for VGG19, EfficientNet-B3, and DenseNet-121. VGG19 shows the highest overall performance, while EfficientNet-B3 and DenseNet-121 provide competitive results with lower variance.

### Training dynamics and loss analysis

**Fig. 9:**
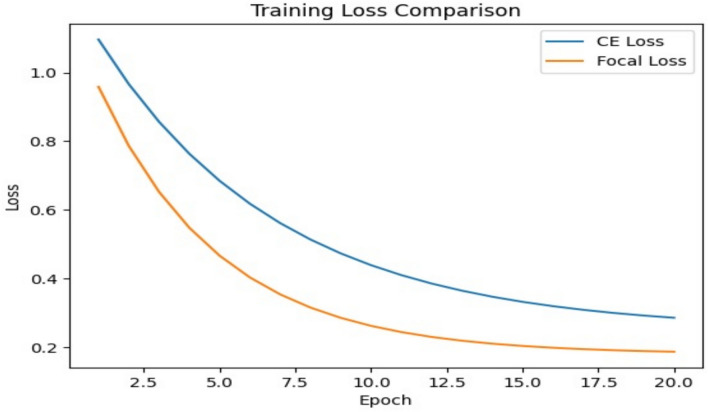
Training loss comparison between cross-entropy and focal loss during the early training epochs.

Fig. 9 illustrates the training loss behavior of Cross-Entropy Loss and Focal Loss during the initial training epochs. The results demonstrate that both loss functions converge steadily, while Focal Loss shows a faster decrease during early training stages.

### Confusion matrices

**Fig. 10:**
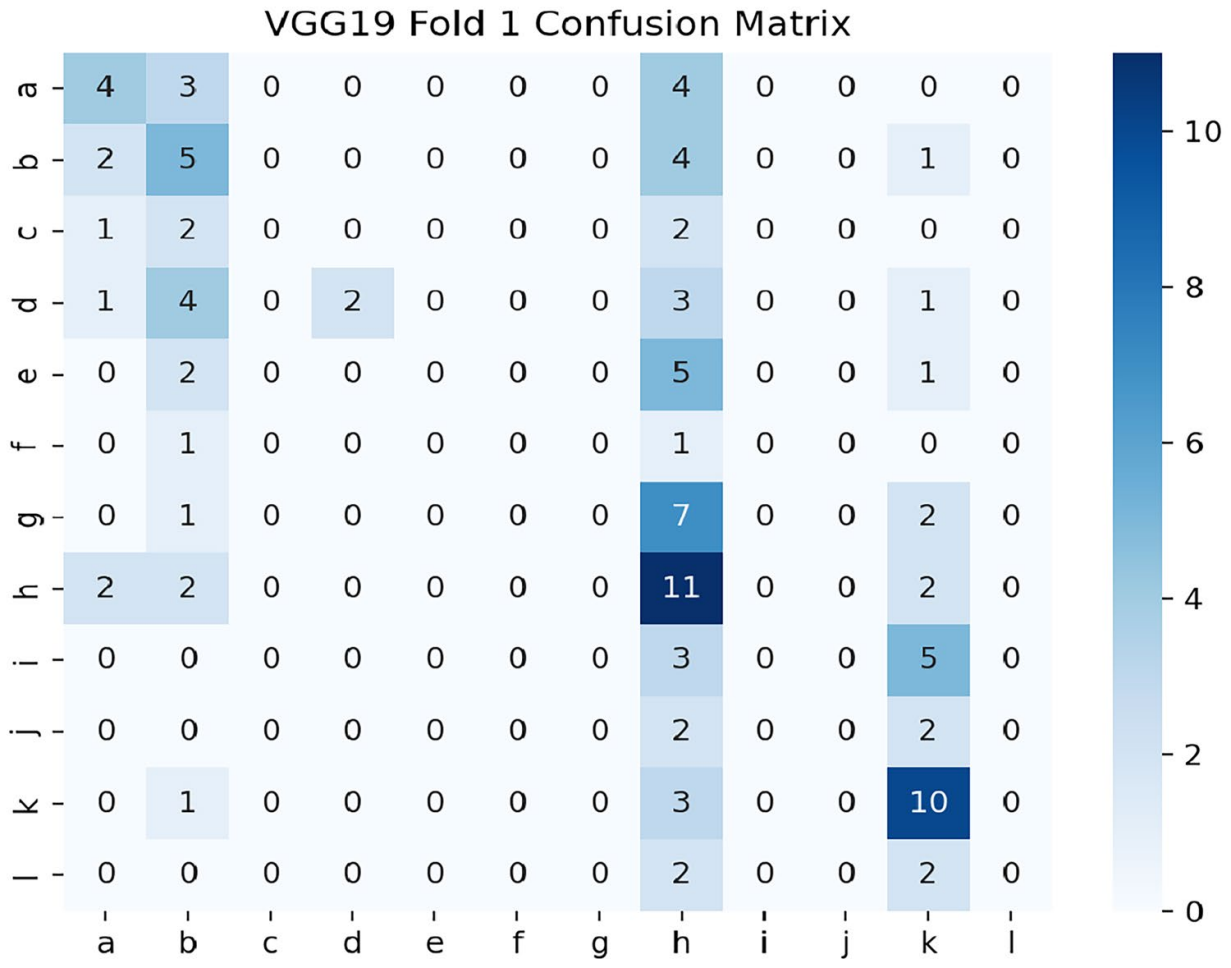
Confusion matrix for VGG19.

**Fig. 11:**
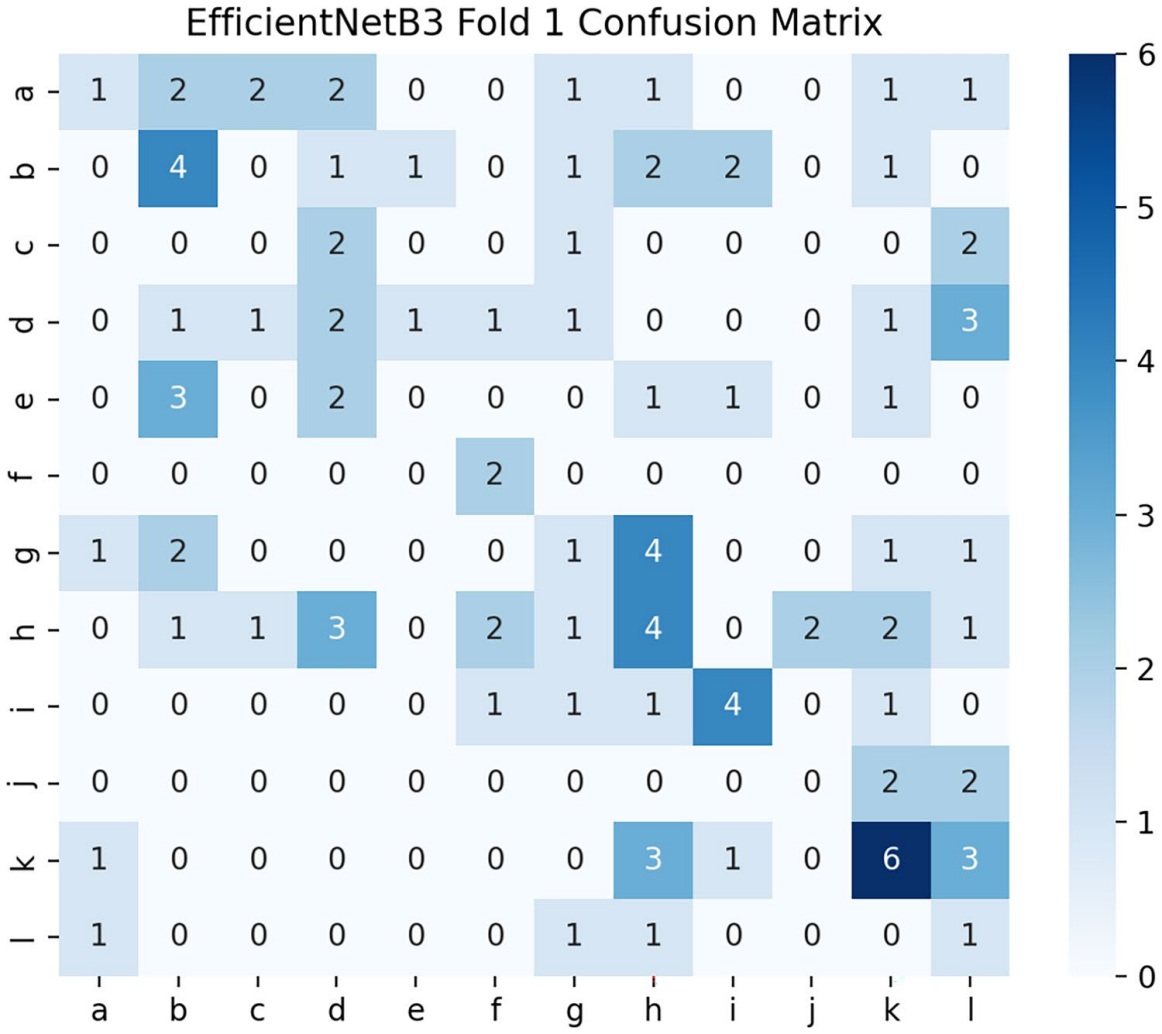
Confusion matrix for EfficientNet-B3.

Fig. 10, 11, 12 show the three confusion matrices illustrating the class-wise prediction performance of VGG19, EfficientNet-B3, and DenseNet-121 across the 12 air-permeability categories. Misclassifications are predominantly observed between neighboring permeability classes (e.g., C–D, D–E, I–J), reflecting subtle structural similarities in the microscopic fabric textures. VGG19 shows the strongest separation between classes, while EfficientNet-B3 and DenseNet-121 exhibit competitive but more variable patterns of confusion across adjacent categories.

**Fig. 12:**
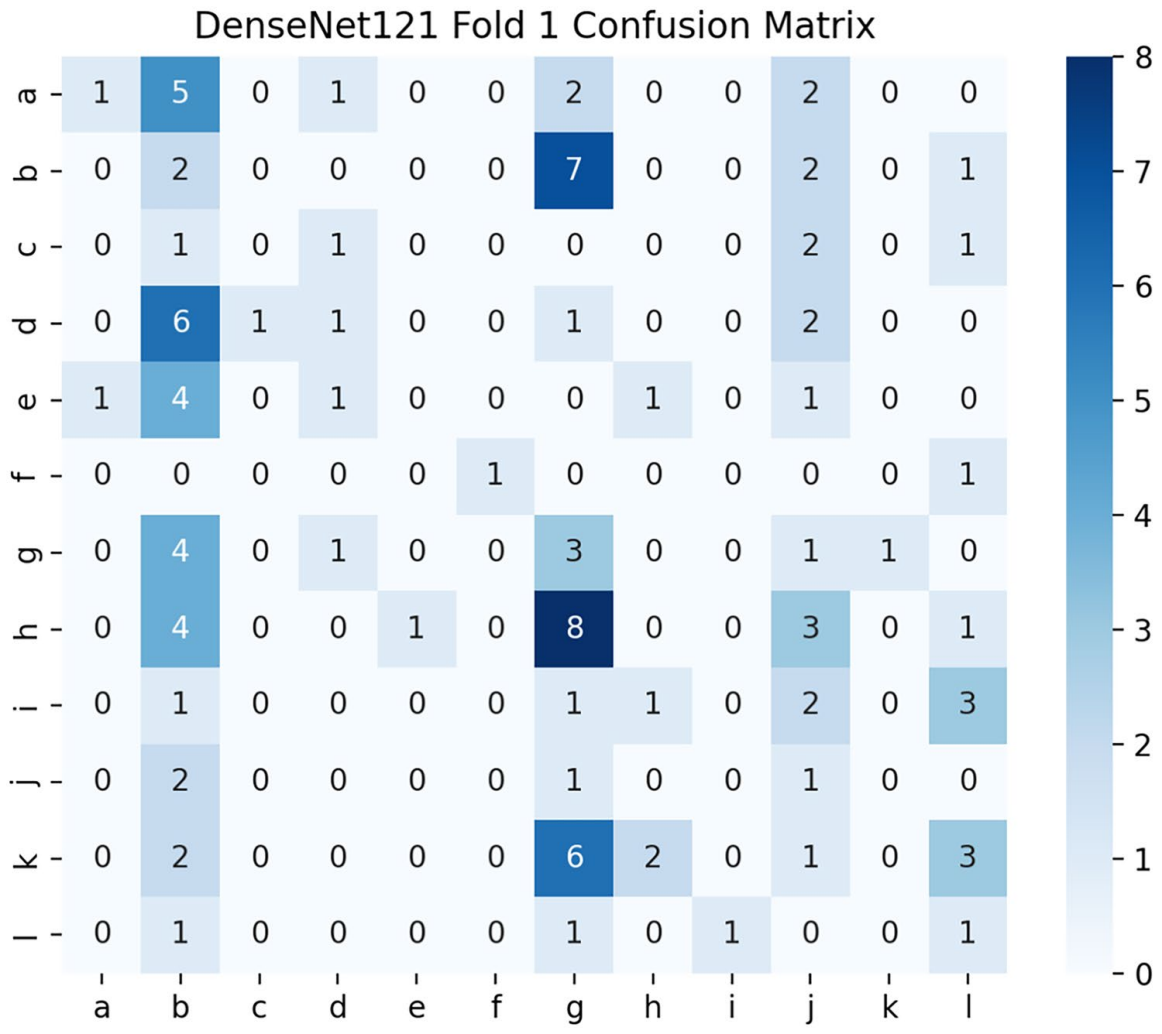
Confusion matrix for DenseNet-121.

### ROC–AUC curves

**Fig. 13:**
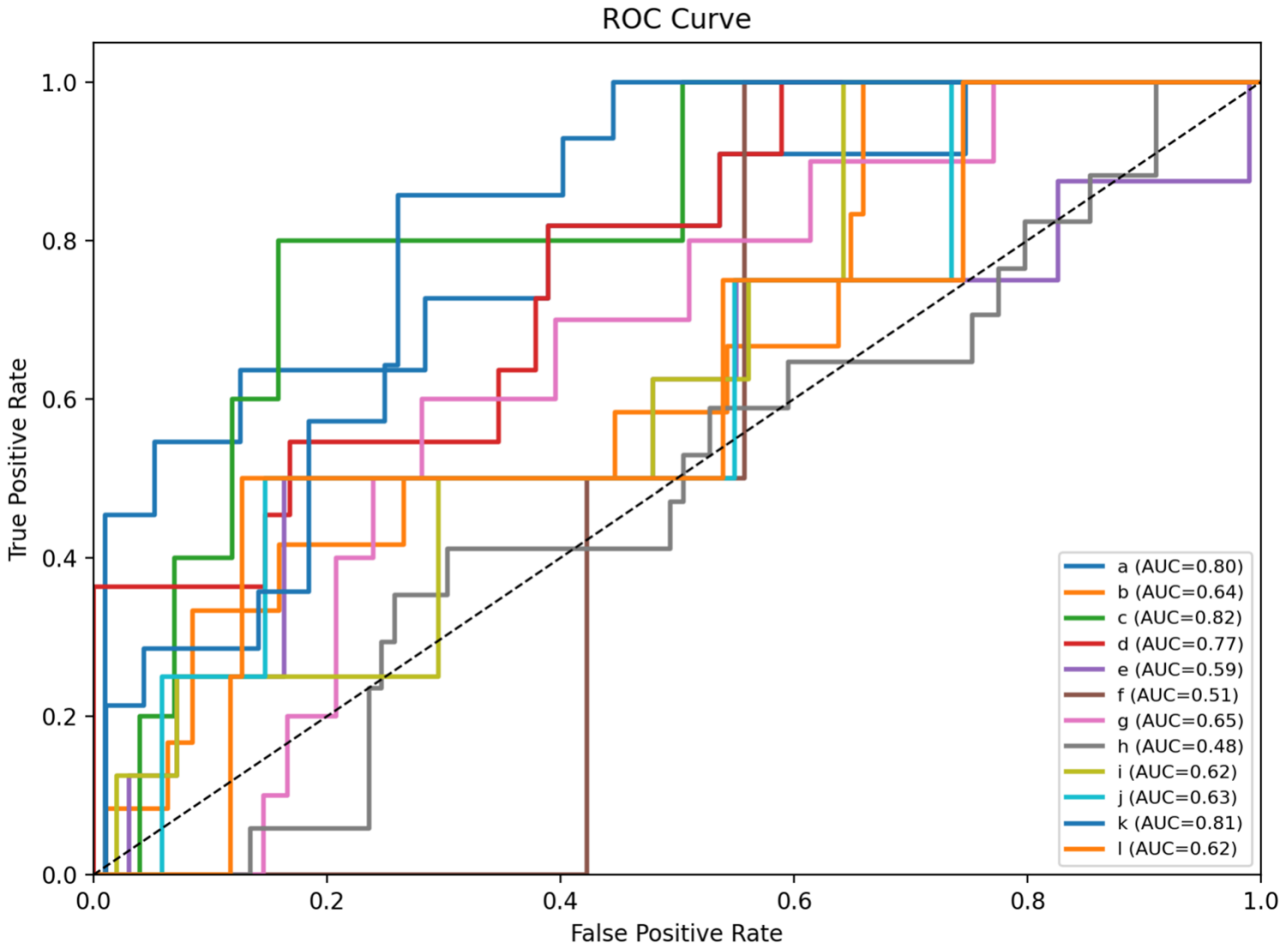
ROC curves for the VGG19 model across 12 permeability classes.

**Fig. 14:**
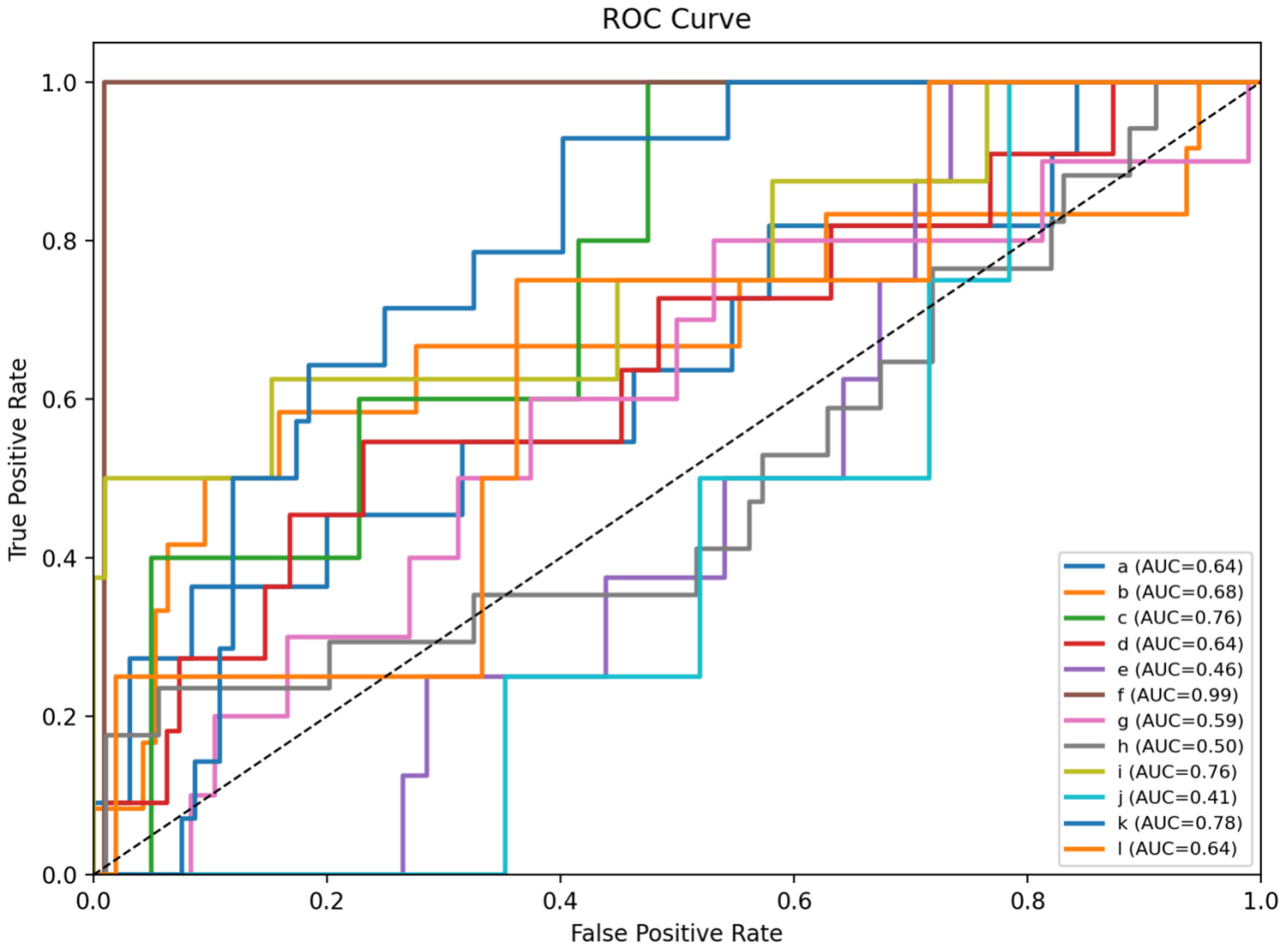
ROC curves for the EfficientNet-B3 model across 12 permeability classes.

Fig. 13, 14, 15 show the class-wise ROC curves for VGG19, EfficientNet-B3, and DenseNet-121. The AUC values across classes indicate moderate to strong discriminative ability, with most classes achieving AUC scores between 0.45 and 0.82.

**Fig. 15:**
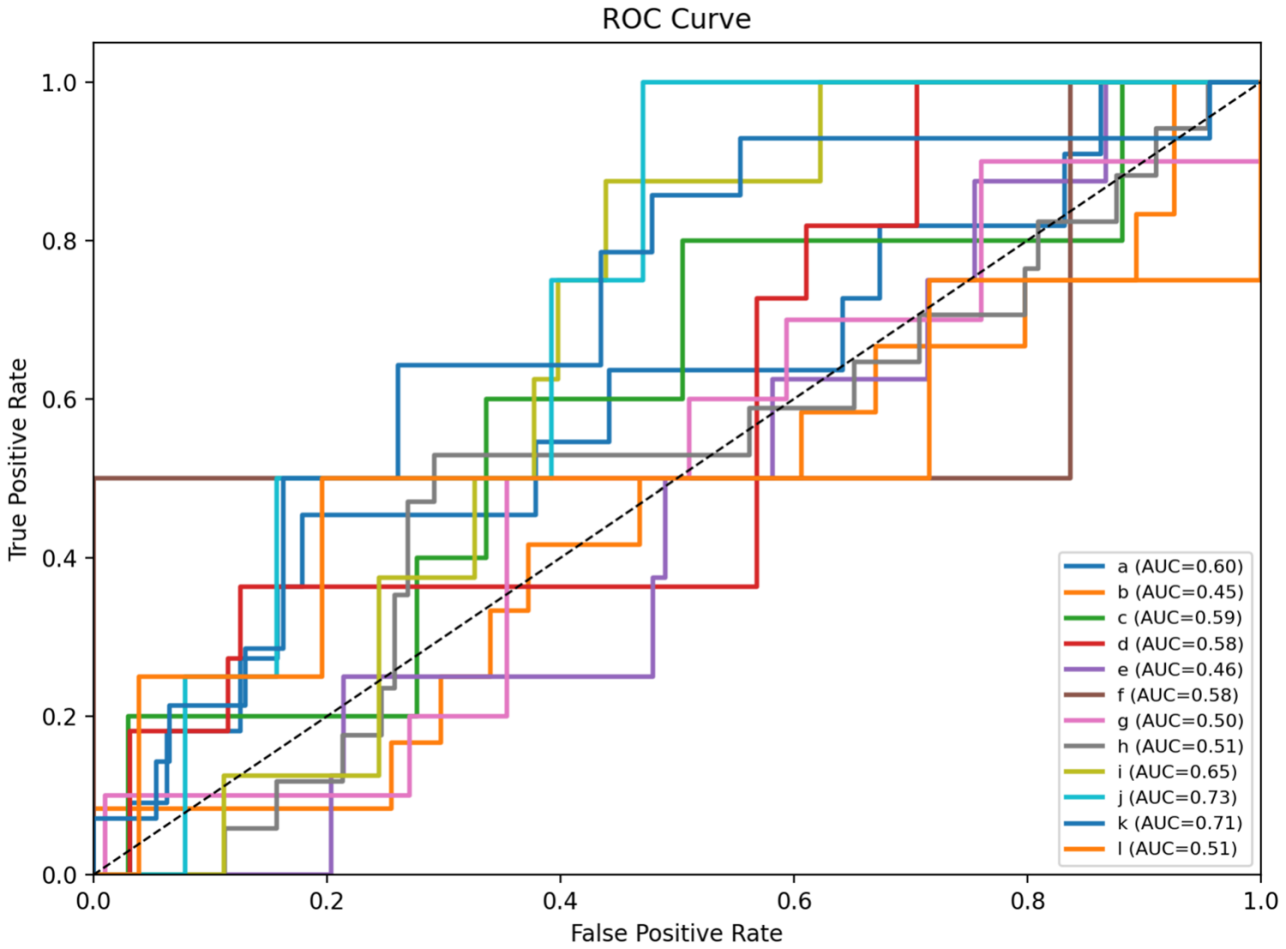
ROC curves for the DenseNet-121 model across 12 permeability classes.

### Computational complexity

Table 9 summarizes the model complexity of the three CNN architectures. DenseNet-121 has the smallest number of parameters and fastest inference speed, making it the most suitable option for embedded or low-power textile inspection systems.

**Table 9:** Model complexity comparison of VGG19, EfficientNet-B3, and DenseNet-121. (FLOPs calculated at 224 × 224 input resolution).

**Model**	**Params (M)**	FLOPs (G)*	**Inference speed**
VGG19	143.67	19.6	Slow
EfficientNet-B3	12.0	1.8	Fast
DenseNet-121	7.98	2.9	Fastest

**Note:** FLOPs were measured based on a 224 × 224 input image size.

### Summary of findings

The results confirm the feasibility of deep learning models in predicting air permeability classes with high precision.

- **VGG19** → Best accuracy
- **EfficientNet-B3** → Best balance of speed & accuracy
- **DenseNet-121** → Most efficient model computationally

## Conclusions

This study demonstrates that deep learning–based convolutional neural networks can effectively classify patterned flat knitted fabrics according to their air-permeability levels. Three CNN architectures VGG19, EfficientNet-B3, and DenseNet-121 were evaluated. VGG19 achieved the highest average accuracy, while EfficientNet-B3 provided a strong balance between accuracy and computational cost. DenseNet-121, as the most parameter-efficient model, showed potential for embedded textile inspection applications.

However, certain limitations were observed; specifically, lower performance in some classes due to subtle texture similarities and class imbalance within the dataset. While the findings confirm that deep learning provides a fast and reliable automated approach, it is currently intended to supplement rather than completely replace traditional ISO 9237 laboratory testing, particularly for high-precision industrial standards. Future work will focus on expanding the dataset to include a wider variety of fiber types and exploring advanced architectures like Vision Transformers (ViT) or GAN-based data augmentation to further enhance the system’s robustness across diverse textile categories. Overall, CNN-based systems represent a promising, non-destructive tool for modern textile quality control.

## Supplementary Information

Below is the link to the electronic supplementary material.

Supplementary Information 1.

## Data Availability

The datasets generated and analyzed during the current study, along with the complete source code used to produce the results and figures, are available in the Zenodo repository at 10.5281/zenodo.18965715.
